# The Response of Rhizosphere Microbial C and N-Cycling Gene Abundance of Sand-Fixing Shrub to Stand Age Following Desert Restoration

**DOI:** 10.3390/microorganisms12091752

**Published:** 2024-08-23

**Authors:** Yunfei Li, Bingyao Wang, Zhanjun Wang, Wenqiang He, Yanli Wang, Lichao Liu, Haotian Yang

**Affiliations:** 1 Key Laboratory of Ecological Safety and Sustainable Development in Arid Lands, Northwest Institute of Eco-Environment and Resources, Chinese Academy of Sciences, Lanzhou 730000, China; liyunfei@nieer.ac.cn (Y.L.); wby@lzb.ac.cn (B.W.); 18298401507@163.com (W.H.); lichao@lzb.ac.cn (L.L.); 2 Shapotou Desert Research and Experiment Station, Northwest Institute of Eco-Environment and Resources, Chinese Academy of Sciences, Lanzhou 730000, China; 3 University of Chinese Academy of Sciences, Beijing 101408, China; 4 Institute of Desertification Control, Ningxia Academy of Agricultural and Forestry Sciences, Yinchuan 750002, China; nxwzhj@163.com; 5 College of Forestry, Gansu Agricultural University, Lanzhou 730070, China; wangyanli126@hotmail.com

**Keywords:** desert ecosystem, soil microbial community, functional genes, C and N cycling, rhizosphere soil

## Abstract

Rhizosphere microorganisms play a pivotal role in biogeochemical cycles, particularly in relation to carbon (C) and nitrogen (N) cycles. However, the impact of stand age on the composition of rhizosphere microbial communities and the abundance involved in C and N cycling remains largely unexplored in restoration ecosystems dominated by shrubs of temperate deserts. This study focuses on revealing changes in microbial composition and functional genes in the rhizosphere soil of *Caragana korshinskii* after revegetation, as well as their response mechanisms to changes in environmental factors. The alpha diversity of bacteria tended to increase with stand age, whereas that of fungi decreased. The abundance of denitrification; dissimilatory nitrate reduction to ammonium, nitrification, and ammonium assimilation; and C fixation-related gene levels increased with stand age, whereas those related to the degradation of starch, pectin, hemicellulose, cellulose, and aromatics decreased. The parameters MBC, MBN, and TC were the key factors affecting the bacterial community, whereas the fungal community was regulated by TN, EC, pH, and MBC. Stand age indirectly regulated C and N cycling functions of genes through altered soil properties and microbial community structures. This study presents a novel approach to accurately evaluate the C and N cycling dynamics within ecosystems at various stages of restoration.

## 1. Introduction

Drylands cover approximately 45% of the terrestrial surface of the Earth. Such arid ecosystems are seriously threatened by increasing desertification caused by human activities and climate change. Ecological restoration is a viable approach to curb and reverse desertification, and the planting of more resistant plants, such as xerophytic shrubs, has been shown to be an important method to restore damaged ecosystems in arid areas. Revegetation not only changes the soil characteristics and the soil microbial community structures [1,2] but also enhances ecosystem services. The restoration of degraded ecosystems via remediation and the improvement of soil quality and soil microbial community diversity is an excellent strategy to minimize the effects of human disturbances on ecological systems. Through natural succession, with the gradual recovery of the vegetation, the characteristics of plant communities and species composition gradually change [3], and environmental factors such as temperature, water, and soil nutrient levels also improve [4]. Previous studies have shown that soil biogeochemical cycles, including carbon (C) and nitrogen (N) cycles, also changed significantly after revegetation [5,6]. However, those series of changes are closely linked to the regulatory mechanisms of soil–plant–microbe interactions.

Rhizosphere microbiomes are a critical part of terrestrial ecosystems and play a significant role both in rhizospheres and bulk soil biogeochemical cycles [2]. They are involved in the regulation of multiple ecosystem processes, and their diversity, structure, and function are highly sensitive to environmental changes. Among others, soil microbial community structures and functions are influenced by soil substrate availability [7], enzyme activity [8], plant attributes [9], and environmental heterogeneity [9]. In addition, the soil microbial community activities are also strongly dependent on soil functions and have unique effects on carbon (C) cycles and nitrogen (N) cycles [10]. On the one hand, the impact of microorganisms on soil C and N cycles is regulated by environmental factors such as soil texture, temperature, moisture, and vegetation type [11]. On the other hand, these impacts are also closely related to the corresponding functional microbial genes [10]. Generally, soil microorganisms obtain their C resources needed for growth mainly by releasing extracellular enzymes that decompose complex organic molecules (such as labile or recalcitrant C) [12,13], whereas their main access to N resources is through the introduction of transporter enzymes to the cell membrane, facilitating the absorption of N-containing compounds produced by processes such as N fixation and nitrification [10]. In these biochemical processes, some crucial microorganisms related to C- and N-cycling will encode a series of extracellular enzymes through C and N cycling genes and subsequently synthesize some specific proteins, thereby promoting soil C and N cycling, such as soil respiration, N mineralization, and immobilization [14]. Consequently, the soil microbial functional genes linked to soil functions are also key regulators of terrestrial C and N cycling [10].

The main site of soil–plant–microbe interactions is the rhizosphere within about 1 mm from the root surface [15]. Plants depend on soil nutrients to complete their growth and development, whereas the exudates released into the rhizosphere during plant growth improve the soil environment [15,16]. According to a previous study, the microbial communities in rhizosphere soils differ significantly from those in bulk soils [17,18]. Root exudates play an important role in the interaction between plants, rhizospheres, and microorganisms and improve the availability of soil nutrients in arid regions [19]. At the same time, as a chemical attractant for microorganisms, exudates can also allow microorganisms to colonize and reproduce in the rhizosphere and root surface, shaping the rhizosphere microbial community [18]. Rhizosphere microorganisms not only influence the structure and succession of bulk soil microbial communities [18,20] but also play a crucial role in regulating plant growth and nutrient uptake [21]. In addition, they play a central role in maintaining processes and functions such as C and N cycling [22]. Given the distinct physicochemical and biological properties of rhizosphere soil compared to bulk soil [22,23], the mechanism governing the impact of the rhizosphere microorganisms on C and N cycling may diverge between these two soil compartments.

Sand-fixing shrubs, as the dominant species in desert ecosystems, are distributed in patches under limited water resources. They possess a substantial root system capable of efficiently utilizing deep soil water reserves; for instance, *C*. *korshinskii* roots can extend several meters deep and occupy a significant spatial area [24]. Previous studies on C and N cycling have primarily focused on bare land or surface soil among shrubs, neglecting the crucial role of rhizosphere microorganisms associated with shrubs [25,26]. This oversight may lead to a serious underestimation of the contribution of shrub rhizospheres to C and N cycling. Previous studies have found that the functional genes and metabolic potential of microbial communities in biological soil crusts and topsoil (0–5 cm) changed markedly with the successional process of revegetation and that changes in microbial community structure determined the relative abundance of functional genes [25]. Additionally, it has been found that bacteria and fungi play a crucial role in the regulation of C and N cycling during dryland revegetation succession and stimulate an increase in soil C and N metabolism in the later stages of revegetation succession [27]. With increasing stand age, deep-rooted shrubs may have a unique effect on deeper soil microbial community structure and abundance of C and N cycling functional genes.

Hence, we hypothesized that: (1) stand age impacts the rhizosphere soil microbial communities, leading to alterations in abundance, diversity, and functional genes related to C and N cycling, and (2) the abundance of C fixation genes may decrease due to the rapid utilization of labile C sources by microorganisms, while N cycling gene abundance increases in response to the greater metabolic demand resulting from the rapid growth and activity of rhizosphere soil microorganisms. Therefore, the main objectives of our study were to (1) examine the changes in rhizosphere soil microbial communities and C and N cycle functional gene abundance along the age gradient of *C. korshinskii* plantation, (2) explore whether changes in soil properties and plant characteristics have different impacts on fungal and bacterial community structures, and (3) distinguish the predominant driving factors of shifts in rhizosphere soil microbial community structures, as well as C and N cycle functional genes along the age gradient of *C. korshinskii* plantation.

## 2. Materials and Methods

### 2.1. Study Area

**Figure 1:**
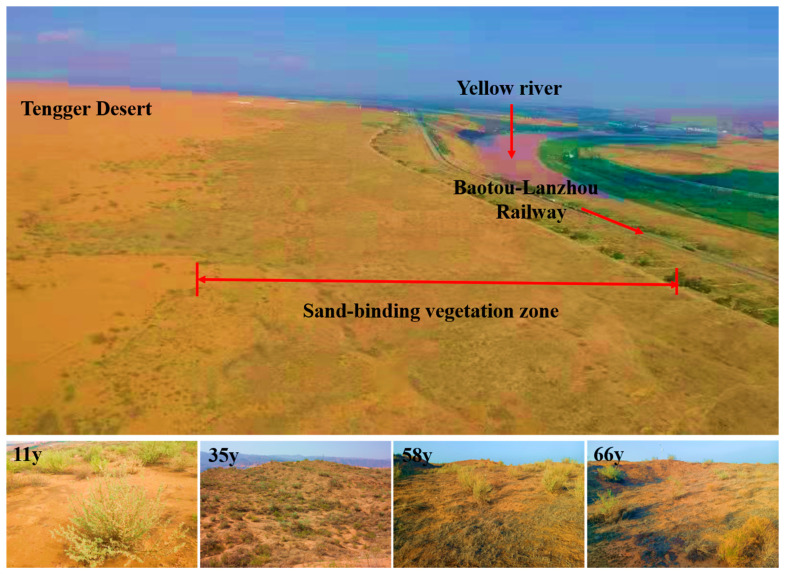
General view of the research sites. 11 y, 35 y, 58 y and 66 y represent vegetation established in 2011, 1987, 1964 and 1956, respectively.

The study area was located in the Shapotou region on the southeastern edge of the Tengger Desert, China (37°25′ N, 105°40′ E, 1330–1350 m above sea level), an ecotone between the steppe and sandy deserts (Figure 1). The annual mean air temperature is 10 °C, with a daily mean air temperature ranging from −9.6 °C in January to 24.3 °C in July. The annual mean precipitation in the area is 186.2 mm, with a mean annual potential evapotranspiration of approximately 3000 mm. The soil is classified as wind-borne sand [24]. There is extremely low natural vegetation cover of less than 1%.

To protect the Lanzhou–Baotou railway from burial by sand, a non-irrigated sand-binding vegetation protection system was constructed from 1956 to 2011. Firstly, straw checkerboard sand barriers (measuring 1 × 1 m) were established. Subsequently, the native shrub seedlings of xerophytic shrubs, such as *Caragana korshinskii* Kom., *Artemisia ordosica* Krasch., and *Hedysarum scoparum* Fisch. were planted within the straw checkerboard at a spacing of 1 m × 2 m or 2 m × 3 m. The vegetated area was further expanded along a south–north direction through the same approach in 1964, 1973, 1987, 1999, and 2011. At present, an ecological recovery area (16 km long and 700 m wide) with a chronosequence is being formed [28]. After over 60 years of natural succession, this system has become a complex artificial–natural composite desert ecosystem combining shrubs and annual herbs. The dominant shrubs in the area were *C. korshinskii* Kom. and *A. ordosica* Krasch. The annual herbs were dominated by *Eragrostis minor* Host, *Setaria viridis* (L.) Beauv., *Chloris virgata* Sw., *Bassia dasyphylla* (Fisch. Et Mey.) O. Kuntze, *Echinops gmelinii* Turcz., and *A. capillaris* Thunb.

### 2.2. Site Selection and Soil Sampling Preparation

Soil samples were collected in July 2022 from the revegetation sites with different stand ages (11, 35, 58, and 66 years) but with similar selected soil types, elevation, slope degree, and aspects. In each of the four sites, three sampling plots (10 × 10 m) were established more than 50 m apart from each other, and across each plot, three to five *C. korshinskii* individuals were investigated randomly to collect the rhizosphere soil. Specifically, the topsoil (0–20 cm) was removed, a transect was established around the plant, and the fine roots (<1 mm) and adhering rhizosphere soil were carefully picked out of the transect with sterile forceps and shaken gently to remove loose soil. Soil adhering to fine roots after gentle shaking was considered rhizosphere soil, whereas the non-adhering soil was defined as bulk soil. The number of fine roots with adhering soil taken on each plot was based on the number needed to collect >50 g of rhizosphere soil. The fine roots with adhering soil were placed in an ice box and immediately transported to the laboratory. In the laboratory, the rhizosphere soil was carefully removed from the fine roots by gently scraping on a sterile operating table. After separation from the fine roots, the rhizosphere soil from the same plot was mixed thoroughly to obtain a composite sample, and a total of 12 composite samples were collected. Each composite sample was divided into three parts: one part was stored at −80 °C, one part was stored at −20 °C, and one part was air-dried for the subsequent analyses.

### 2.3. Determination of Soil Physicochemical Properties

The total C (TC) levels of the rhizosphere soil were determined using a TOC analyzer (Vario II, Elementar, Langenselbold, Germany), and the total nitrogen (TN) contents were measured with an element analyzer (Vario MACRO cube, Elementar Inc., Germany). The concentrations of available phosphorus (AP) were measured using the standard methods of the Chinese Ecosystem Research Network (CERN). Soil pH was determined in a soil–water mixture with a ratio of 1:5 using a pH meter (CRISON 20, Barcelona, Spain), and electrical conductivity (EC) was measured with a portable conductivity meter (Cole-Parmer Instrument Company, Vernon Hills, IL, USA). The microbial biomass carbon (MBC) and microbial biomass nitrogen (MBN) concentrations were determined by the chloroform fumigation–potassium sulfate extraction method. The activities of *β*-1,4-glucosidase (BG), alkaline phosphatase (ALP), and *β*-N-acetylglucosaminidase (NAG) were determined fluorometrically using two solutions of substrates labeled with 4-methylumbelliferone as well as 7-amino-4-methylcoumarin [29,30].

### 2.4. Shotgun Metagenome Sequencing and Sequence Processing

The DNA was extracted from rhizosphere soil samples using the CTAB method [31]. The DNA degradation degree, potential contamination, and concentration were measured using the software Agilent 2100 (Agilent Technologies Co. Ltd., Santa Clara, CA, USA) to screen qualified samples for sequencing. Sequencing libraries were generated using the NEBNext^®^ Ultra™ DNA Library Prep Kit for Illumina (NEB, San Diego, CA, USA) following the manufacturer’s recommendations, and index codes were added to attribute sequences to each sample. The index-coded samples were clustered on a cBot Cluster Generation System [32] according to the manufacturer’s instructions. After clustering, the library preparations were sequenced on an Illumina Novaseq 6000 platform, and 2 × 150-bp paired-end reads were generated. To ensure data reliability, the raw sequencing data were filtered with Trimmomatic to obtain high-quality clean reads [33]. The reads of host origin in the clean data were filtered using the Bowtie 2 software to eliminate the possibility of host pollution. The clean reads after quality control and de-hosting were used for database BLAST (Uniref90) employing the Humann2 software. Kraken 2 was employed to identify the species contained in the samples through comparison with the NCBI database, and subsequently, the actual relative abundance of species in the samples were predicted using Bracken [34]. The microbial profiles of function were generated by HUMAnN3 according to the corresponding relationship between the Uniref90 ID and KEGG database [35].

### 2.5. Carbon and Nitrogen Functional Gene Screening from Shotgun Metagenome Sequence

The pathways of map00624 and map00710 in the KEGG database were screened as major C degradation and C fixation pathways in rhizosphere soil. The 10 pathways included in the N metabolism pathway (map00910) were screened from the KEGG database. Subsequently, we screened all functional genes involved in these pathways from the shotgun metagenome data in this study, namely, those genes related to the carbon and nitrogen cycles. We classified all the genes in the map00627 pathway into seven categories based on the substrate differences for each functional gene, namely, functional genes involved in the degradation of starch, pectin, hemicellulose, cellulose, aromatics, chitin, and lignin.

### 2.6. Statistical Analysis

One-way ANOVA following Tukey HSD at *p* < 0.05 was used to investigate the effects of stand age on soil properties and the abundance of genes involved in C and N cycling. The non-metric multidimensional scaling (NMDS) based on Bray–Curtis distances was performed to visualize C and N cycling microbial community composition at the phylum level. Redundancy analysis (RDA) was used to test the relationship between the soil microbial community structure and environmental factors. Heatmaps were employed to investigate the link between soil biotic/abiotic factors and the abundance of microbial functional genes. To reveal the influential factors of the functional gene abundance involved in C and N cycling, we applied Mantel tests to evaluate the correlations between gene abundance and bio-abiotic drivers. Variation partitioning analysis (VPA) was also used to assess the effect of soil and microbial properties on microbial functional genes involved in C and N cycling. All data analysis was performed with R 4.1.3.

To determine the direct and indirect effects of stand age on the abundance of soil microbial C and N cycling genes, we constructed and tested a partial least squares path modeling (PLS-PM) model. Firstly, we constructed a full PLS-PM model involving all the available variables based on known and potential relationships among different variables. We then removed the less significant variables until we attained the final model. Goodness-of-fit PLS models were evaluated using the goodness-of-fit (GOF) statistic. The PLS-PM model was performed using SmartPLS 3.0 [36].

## 3. Results

### 3.1. Characteristics of Soil Abiotic and Biotic Factors

Significant differences were found in soil properties among the four stand ages (*p <* 0.05; Table 1). The contents of TN, TC, AP, EC, MBC, and MBN in the rhizosphere soil significantly increased with stand age (*p <* 0.05). The levels of ALP and NAG also increased with stand age, but the differences were not statistically significant (*p >* 0.05). At 66 years of revegetation, the respective values were 149.38%, 83.26%, 15.68%, 486.22%, 235.25%, 202.63%, 6.96%, and 34.23%, respectively (Table 1). However, the pH and BG levels significantly decreased with stand age (*p <* 0.05) and were 3.39% and 26.75% lower, respectively, after 66 years (Table 1).

### 3.2. Soil Microbial Community Composition Changes along Stand Age

**Figure 2:**
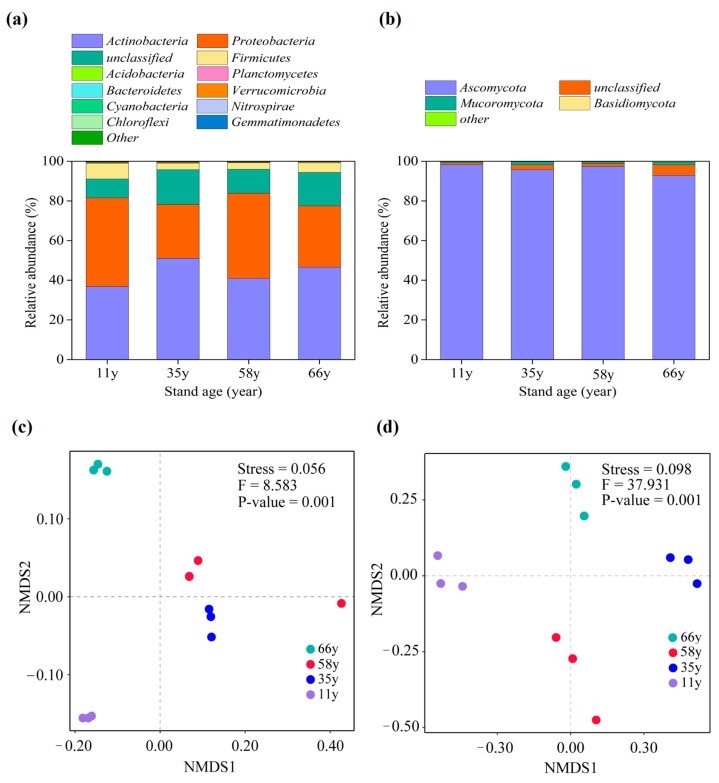
The change of soil microbial communities’ composition along stand age. (**a**) Relative abundance of dominant bacterial phyla; (**b**) relative abundance of dominant fungal phyla; (**c**) NMDS of bacterial phyla; (**d**) NMDS of fungal phyla. “Other” includes phyla with a relative abundance < 0.1% at the class level.

Based on the results, the soil bacterial and fungal community composition in the phyla level and genus level changed significantly along stand age (Figure 2 and Figure S1). The dominant bacterial phyla were Actinobacteria, Proteobacteria, unclassified, and Firmicutes (relative abundance > 1%), of which Actinobacteria and unclassified showed increasing abundance with stand age, whereas Proteobacteria and Firmicutes showed the opposite pattern (Figure 2a). Regarding the soil fungal community, Ascomycota, unclassified, and Mucoromycota were the dominant phyla (relative abundance > 1%), of which Ascomycota had a large relative abundance in the 11-year-old site, which then decreased with stand age, whereas unclassified and Mucoromycota showed the opposite change trend (Figure 2b). At the genus level of categorization, the bacterial community was dominated by Streptomyces, Sinorhizobium, Bradyrhizobium, Neorhizobium, Mesorhizobium, Jiangella, Rhizobium, Priestia, Pseudonocardia, Geodermatophilus, Nocardioides, Variovorax, and Arthrobacter (relative abundance > 1%), and the fungal community was dominated by Penicillium, Fusarium, Aspergillus, and Purpureocillium (relative abundance > 1%) (Figure S1).

The Shannon index of bacteria was highest in the 11-year-old site and decreased with stand age. In contrast, the Shannon index of fungi was lowest in the 11-year-old site and tended to increase with stand age (Figure S2). To understand the soil bacterial and fungal changes, the soil bacterial and fungal communities were clustered and aggregated according to nonmetric multidimensional scaling (NMDS). Based on the results, the soil microbial community composition significantly differed among stand ages (*p* = 0.001), and the patterns of *β*-diversity based on species composition were correlated with soil microbial community type (Figure 2c,d). In particular, for soil bacterial phyla, the microbial communities appeared to be clustered in the 35- and 58-year-old sites and separate in the 11- and 66-year-old sites (Figure 2c). However, regarding the soil fungal phyla, the microbial communities appeared to be separate in the 11-, 35-, 58-, and 66-year-old sites (Figure 2d).

### 3.3. Soil Microbial Functional Gene Abundance along Stand Age

**Figure 3:**
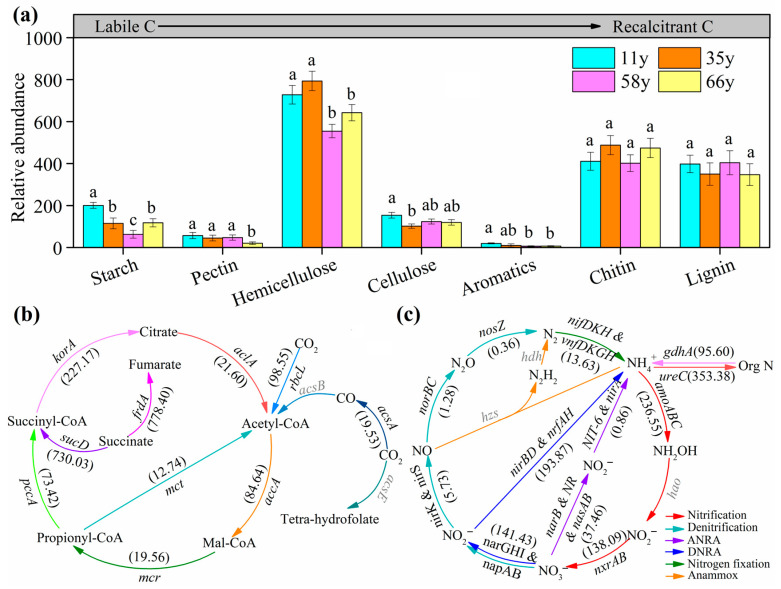
The relative abundance of soil microbial functional gene of carbon and nitrogen cycling with stand age. (**a**) Carbon degradation genes; the different lowercase letters indicate significant differences at *p* < 0.05 level between the different stand age. (**b**) carbon fixation genes; different colored arrows represent different genes. (**c**) nitrogen cycling genes, different colored arrows represent different nitrogen metabolism pathways.

We analyzed 34 gene families that encoded enzymes related to C degradation, classified according to the metabolized compound types, including starch, pectin, hemicellulose, cellulose, chitin, aromatics, and lignin (Figure 3a and Figure S3). Genes involved in the degradation of hemicellulose, chitin, and lignin were the dominant genes. We observed that with the age of the stand, the relative abundance of genes involved in the degradation of labile C (such as starch, pectin, hemicellulose, cellulose, and aromatics) decreased significantly, whereas the relative abundance of genes involved in the degradation of recalcitrant C (such as chitin, lignin) had no significant difference between different ages (Figure 3). Moreover, stand age also has a significant influence on the relative abundance of genes involved in C fixation. Within the Calvin cycle, the abundance of succinate pathway genes (*frdA*, *sucD*) takes precedence (Figure 3b). The relative abundance of *accA*, *pccA*, and *rbcL* genes gradually decreases with stand age, whereas the relative abundance of *aclA*, *frdA*, *korA*, *mcr*, *mct*, and *sucD* genes showed a trend of first increasing and then slightly decreasing with stand age (Table S1).

Among the N cycling process, the relative abundance of nitrification, denitrification, assimilatory nitrate reduction (ANRA), dissimilatory nitrate reduction (DNRA), nitrogen fixation, anammox, mineralization, and ammonium assimilation genes all changed significantly with stand age (Figure 3c, Table S1). The relative abundance of nitrification (*amoABC*, *nxrAB*), DNRA (*narGHI & napAB*, *nirBD & nrfAH*), mineralization (*gdhA*), and ammonium assimilation (*ureC*) process genes were dominant (Figure 3c). The relative abundance of genes associated with denitrification (*narGHI* and *napAB*), DNRA (*narGHI* and *napAB*), and nitrification (*nxrAB*) first increased significantly and then decreased slightly with stand age, whereas those of genes associated with nitrification (*amoABC*) and ammonium assimilation (*ureC*) significantly increased with stand age (*p* < 0.05, Table S3).

### 3.4. Impacts of Environmental Variables on Rhizosphere Soil Microbial Community Composition and Functional Gene Abundance

**Figure 4:**
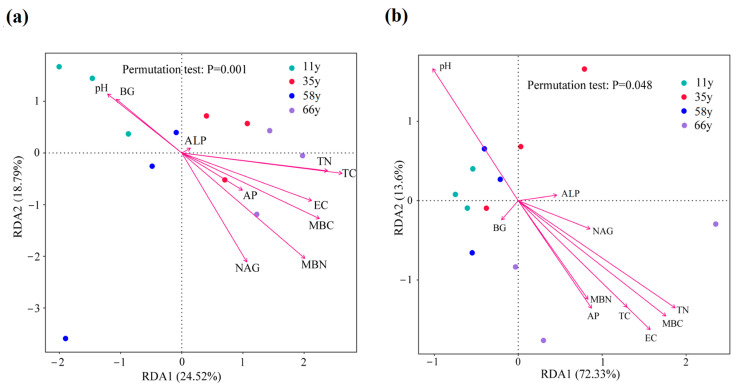
Redundancy analysis showing the relationship between soil microbial community (at a phylum level) and environmental factors. TN, total nitrogen; TC, soil total carbon; AP, available phosphorus; EC, electrical conductivity; MBC, microbial biomass carbon; MBN, microbial biomass nitrogen; BG, *β*-1,4-glucosidase; ALP, acid phosphatase; NAG, *β*-N-acetyl glucosaminidase. (**a**) Bacteria; (**b**) fungi.

The RDA results indicated that the bacterial and fungal community compositions were closely linked to the changes in soil biotic and abiotic factors (Figure 4). Axes 1 and 2 explained 24.52% and 18.79%, respectively, of the variations in the bacterial community (Figure 4a). Based on the results of the permutation test, MBC (R^2^ = 0.57, *p* = 0.02), MBN (R^2^ = 0.64, *p* = 0.007), and TC (R^2^ = 0.59, *p* = 0.02) were the main factors explaining the bacterial community variations. Axes 1 and 2 explained 72.33% and 13.60%, respectively, of the variations in the fungal community (Figure 4b). The permutation test analysis results showed that TN (R^2^ = 0.86, *p* = 0.001), EC (R^2^ = 0.84, *p* = 0.0005), pH (R^2^ = 0.72, *p* = 0.002), and MBC (R^2^ = 0.85, *p* = 0.0005) were the key factors affecting the fungal community structure.

**Figure 5:**
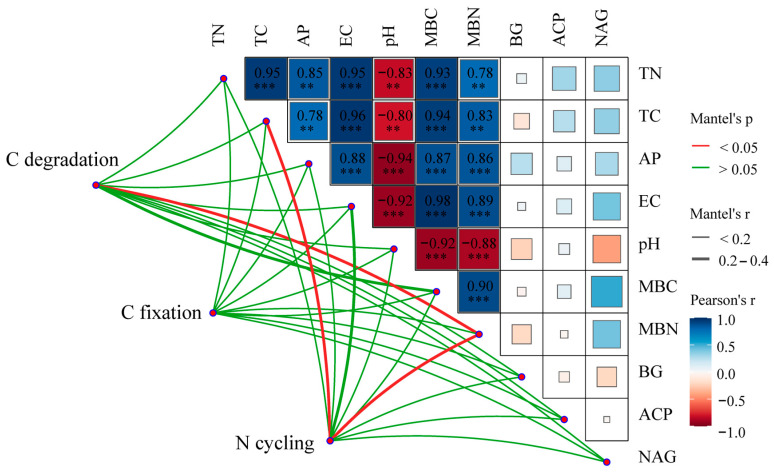
The correlation of environmental factors and carbon and nitrogen cycling functional genes. Edge widths corresponding to Mantel’s r statistic indicate the corresponding distance correlation, and edge color indicates statistical significance. For each panel, the color is proportional to the Pearson’s correlation coefficients (dark blue, r = − 1, dark red, r = 1) (*** indicates *p* < 0.001; ** indicates *p* < 0.01).

The functional genes associated with C degradation and N cycling were correlated with soil properties, MBC, MBN, and soil enzyme activities (Figure 5). The Mantel test results showed that the relative abundance of C degradation genes was significantly correlated with MBN, whereas the relative abundance of N cycle genes was significantly correlated with MBN and TC (*p* < 0.05, Figure 5). There are significant positive correlations between TN, TC, AP, EC, MBC, and MBN, while pH has a significant negative correlation with them. The relative abundance of C degradation genes (such as *sga*, *RgaE*, *abnA*, *xynB*, *CDH*, *E3.2.1.14*, *E3.2.1.4*, and *moxA*) were positively correlated with BG (*p* < 0.05), and those of *E3.2.1.15*, *rgl*, *ara*, *abnA*, *GUSB*, and *moxA* were negatively correlated with soil TC, TN, EC, MBC, and MBN (*p* < 0.05). The relative abundance of C fixation genes (such as *acsA*, *pccA*) has a significant positive correlation with BG, whereas those of *accA*, *frdA*, and *korA* were negatively correlated with TC, TN, EC, MBC, MBN, BG, and NAG (*p* < 0.05). Regarding the genes involved in N cycling, the relative abundance of *narGHI*, *napAB*, *nxrAB*, *amoABC*, *gdhA*, and *ureC* was positively correlated with soil TN, TC, EC MBC, and MBN (*p* < 0.05), while *norBC*, *gdhA*, *ureC*, *nifDKH*, and *vnfDKGH* were negatively correlated with soil TN, C:N, and NAG (*p* < 0.05, Figure S4).

**Figure 6:**
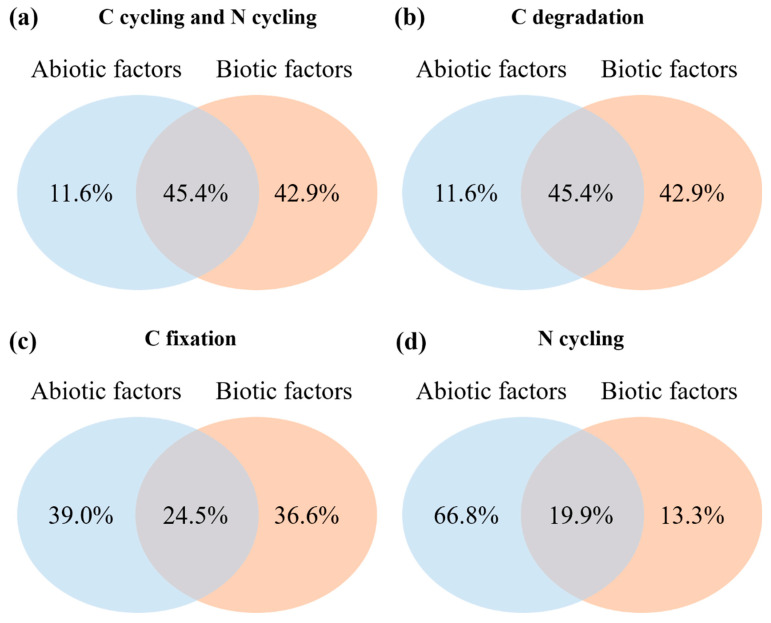
Variance partitioning analysis of the individual and combined explanatory power of abiotic and biotic factors on the relative abundance of carbon and nitrogen cycling functional genes. (**a**) carbon cycling and nitrogen cycling genes; (**b**) carbon degradation genes; (**c**) carbon fixation genes; (**d**) nitrogen cycling genes.

In addition, we found a great difference in the level of explanation of genes involved in C and N cycling based on biotic and abiotic factors (Figure 6). Overall, the explanation ratio of the biotic factors to the abundance of C and N cycle genes was higher than that of the abiotic factors (Figure 6a). The contribution rate of biotic factors to the relative abundance of C degradation genes is 42.9%, while the contribution rate of abiotic factors to the relative abundance of C degradation genes is 11.6%, and the interaction between biotic factors and abiotic factors can explain 45.4% of the variation (Figure 6b). In contrast, abiotic factors contributed more to the relative abundance of C fixation and N cycling genes than biotic factors (Figure 6c,d). The abiotic factors can account for 39.0% and 66.8% of the variation in the relative abundance related to C fixation and N cycling genes, respectively, whereas the biotic factors can account for 36.6% and 13.3% of the variation in the relative abundance related to C fixation and N cycling, respectively, and the interaction between biotic factors and abiotic factors can explain 24.5% and 19.9% of the variation, respectively (Figure 6c,d).

**Figure 7:**
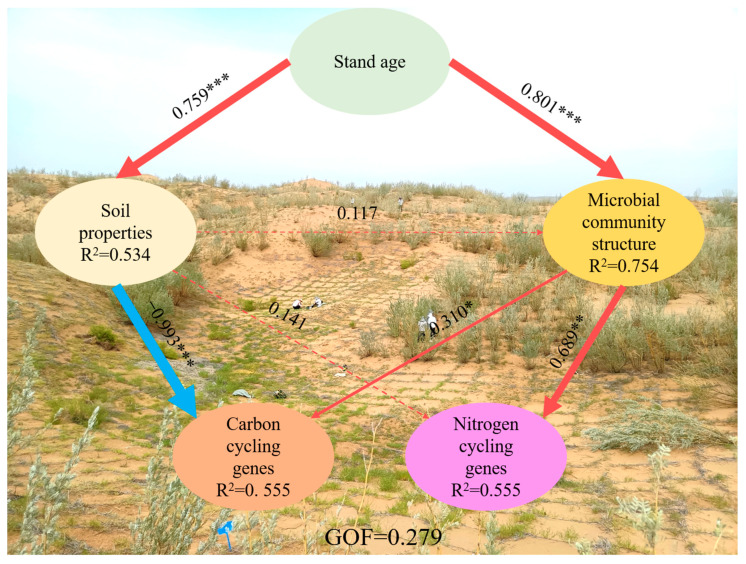
The direct and indirect effects of environmental factors on soil microbial carbon and nitrogen cycling functional genes using partial least squares path models (PLS-PM). The width of the arrows is proportional to the strength of the causal relationship, and the numbers are the correlation coefficients. Red solid lines indicate positive relationships and blue solid lines indicate negative relationships. R^2^ values represent the explained variance of each factor. The solid lines represent significant (*** *p* < 0.001, ** *p* < 0.01, * *p* < 0.05) and the dashed lines represent non-significant (*p* > 0.05).

In order to evaluate the impact of stand age on soil properties, microbial community structure, and C and N cycle functional genes, we applied a PLS-PM model to understand what factors influence the relative abundance of soil microbial functional genes (C and N cycles). Stand age directly drives changes in soil properties and microbial community structure, thereby affecting changes in the relative abundance of soil microbial C and N cycling genes (Figure 7). The negative effect of soil properties (path coefficient = −0.993, *p* < 0.001) was greater than the positive effect of microbial community structure (path coefficient = 0.310, *p* < 0.05) on the soil microbial C cycling genes. In addition, there was a positive effect of microbial community structure on soil microbial N cycling genes (path coefficient = 0.689, *p* < 0.01), which was greater than the direct effects from soil properties (path coefficient = 0.141). The positive effect of stand age (path coefficient = 0.801, *p* < 0.001) on the microbial community structure was greater than the effects of soil properties (path coefficient = 0.117). Furthermore, there was a positive direct effect of stand age on soil properties (path coefficient = 0.759, *p* < 0.001) (Figure 7). The final model explained 53.4% of the variation in soil properties, 75.4% of the variation in microbial community structure, 55.5% of the variation in the relative abundance of soil C cycling genes, and 55.5% of the variation in the relative abundance of soil N cycling genes (Figure 7).

## 4. Discussion

### 4.1. Effects of Stand Age on Soil Properties

Revegetation can effectively improve soil nutrient levels and has an important effect on soil properties [2,25]. In this study, an increasing stand age resulted in significantly improved soil abiotic and biotic factors (Table 1), and the PLS-PM model showed that stand age explained 53.4% of the variation in soil properties (Figure 7). The TC content of the rhizosphere soil gradually increased with stand age, mainly because the exudates of the deep-rooted xerophytic shrubs substantially contributed to organic matter input, facilitating TC accumulation [37]. The TN content increased significantly with stand age, which could be attributed to the N fixation of legume plants; *C. korshinskii* is a shrub species commonly used for ecological restoration in arid and semiarid areas. N-fixing legumes can effectively enhance the restoration of degraded soils in desert regions [38], whereas the bioavailability of P in desert soils is low because of the low solubility of phosphate minerals at neutral to alkaline pH values. The main direct source of P is the decomposition of soil organic matter. In this study, vegetation restoration significantly increased the AP content in rhizosphere soil, which was closely related to the accumulation of root exudates in the soil. According to a previous study, oxalic acid in root exudates can effectively increase the AP level [39,40]. With increasing stand age, the EC showed a gradually increasing trend, whereas the pH decreased. These observations are closely linked to the accumulation of root exudates in the rhizosphere soil, which can affect the EC by regulating the soil microbial community composition, the soil pH, and the osmotic pressure of the soil solution [41]. In addition, root exudate input can indirectly affect the EC through DOC or through its effect on soil properties (mainly soil pH, aFe, and actinomycetes) [41]. Organic acids, protons, and inorganic ions in the root exudates play an important regulatory role in root-soil pH and oxidation-reduction potential [42,43]. All of these changes have contributed to positive changes in the soil environment, and soil ecological functioning has gradually evolved for the better under the continuing influence of plant and microbial interactions.

Most microorganisms in the soil are heterotrophic and absorb exogenous nutrients, consuming energy to maintain their life activities [2]. The accumulation of soil nutrients (mainly from root exudates) provides large amounts of available substrates for the growth and colonization of microorganisms, thereby attracting microorganisms in the rhizosphere soil [44]. In this study, both the MBC and MBN levels increased significantly with stand age, with MBC and MBN levels over 66 y being 3.35 and 3.03 times higher than 11 y, respectively. This can be explained by the variety of shrub roots, which increased with stand age, thus forming a dense root system. Large amounts of root exudates and rotten roots provide sufficient energy for microbial metabolism and self-synthesis. As a result, the nutrient limitation of microbial processes is diminished and microorganisms are able to reproduce in large numbers. Additionally, enzyme activities play an important role in soil nutrient cycling and can therefore be used as indicators of the level of soil fertility and microbial activity [2]. The soil enzyme activities were closely linked to the decomposition of organic compounds and soil microbial resource use limitation as the transformation of important organic elements is promoted by microorganisms [45,46]. In our study, both the ALP and NAG levels increased significantly with stand age, whereas the BG level decreased (Table 1). This suggested that the availability of soil P and N may limit the activity of microorganisms in the rhizosphere soil rather than C, as the microbial nutrient limitation hypothesis suggests that high enzyme activity targeting a specific resource indicates the nutrient limitation of that resource [46], which can be attributed to the fact that the growth and reproduction of microorganisms require more AN and AP. Thus, more ALP and NAG are produced by microorganisms to degrade organic matter to obtain P and N. However, root exudates provide large amounts of available C (such as DOC) [41,47], thus reducing or even eliminating the C limitation for microbial growth, and consequently, microorganisms minimized the synthesis and release of enzymes related to the acquisition of carbon resources, which explains the downward trend of the BG level.

### 4.2. Effects of Stand Age on the Soil Microbial Community and the Genes Related to C and N Cycling

Generally, ecosystems are stable when the soil biodiversity is high. In this study, the soil microbial richness was significantly affected by stand age, and NMDS showed that the community structure characteristics of each site were separated from each other (Figure 2c,d). This suggests that stand age increased and long-term vegetation restoration resulted in different evolution characteristics of bacteria and fungi in the rhizosphere soil. Additionally, the soil bacterial community was dominated by the phyla Proteobacteria and Actinobacteria, consistent with the results obtained for different soil environments [2,48,49]. The phylum Proteobacteria is one of the largest phyla of soil bacteria and not only contains many pathogenic bacteria but also many bacteria with N-fixing functions (such as rhizobia). Actinobacteria are often the dominant bacteria in terrestrial ecosystems [50]. They are ubiquitous and can also be found in extreme ecosystems, making them one of the major components of the soil microbiota. The phylum Actinobacteria contains numerous saprophytic bacteria, whose main function is to promote the decomposition of organic residues in the soil and produce various secondary metabolites [51]. In this study, stand age significantly changed the relative abundance of Proteobacteria and Actinobacteria, the increase in the abundance of Actinobacteria with increasing stand age indicates that improve in the transformation ability of organic matter by soil microorganisms in the later stages of revegetation. Additionally, the changes in microbial community composition were closely related to the level of TC (Figure 4). The TC increased significantly with stand age, providing a favorable environment for the survival of Proteobacteria and Actinobacteria. Furthermore, Actinobacteria are also copiotrophic microbes and enriched in nutrient-rich soils; they play an important role in soil C cycling [52], and the gradual increase in the abundance of Actinobacteria along stand age in our study is consistent with the accumulation of TC with revegetation. Equally, this indicates that soils are also forming from nutrient-poor to nutrient-rich with increasing stand age.

Vegetation restoration and reconstruction regulate the interaction between microbial communities and vegetation succession, mainly manifested as dynamic changes in microbial diversity and structure [53,54]. In this study, the soil fungal community diversity and structure responses to stand age were investigated, and the results are in agreement with previous findings [54,55]. We used the PLS-PM model to assess the direct and indirect effects of stand age on microbial community structure. The results showed that stand age can not only directly affect microbial community structure but also indirectly regulate microbial community structure through soil properties (Figure 7). The Shannon index showed a gradually increased trend in fungi with stand age due to two reasons. First, the native fungi gradually adapted to the environment with increasing stand age [54]. Second, as the stand age increases, the accumulation of organic matter from root exudates and debris provides a more diverse range of habitats and substrates for soil fungal communities, thereby promoting fungal diversity [54]. This indicates that the revegetation of *C. korshinskii* in desert areas influenced the soil fungal alpha diversities, and the increase in diversity confirmed that the increase in stand age plays a positive role in regulating the development of soil fungal communities in revegetated desert ecosystems [54].

Understanding the responses of functional groups to stand age is fundamental to accurately predicting the potential impacts of revegetation on ecological processes. The variations in functional gene abundance among the different stand ages in this study were directly linked to soil properties and microbial community structure (Figure 7 and Figure S4). The relative abundance of most functional genes involved in C degradation decreased with stand age, such as those of *amyA*, *cdaR*, *SGS1*, *rgl*, *ara*, *xylA*, *pssA*, *aceB*, *glx*, and *moxA*, which are important genes involved in the degradation of starch, pectin, hemicellulose, cellulose, chitin, aromatics, and lignin [52]. This result is a perfect explanation for the negative correlation between soil properties and C cycling genes in the PLS-PM model (Figure 7) because the C cycling genes in the model are composed of cellulose, chitin, lignin, and pectin (Figure S5). These findings can be explained by the theory of “nutrient limitation”, which implies that microbial succession is often limited by nutrient availability [56]. Microbial growth in the 11-year-old site was primarily limited by soil nutrient availability, and thus the microorganisms had a high abundance of C-degrading genes to regulate the production of relevant soil extracellular enzymes, which in turn would be used to obtain sufficient nutrients [57]. The quantity and quality of organic matter originating from root exudates are closely related to stand age [58]. With increasing stand age, the accumulation of organic matter in the soil can provide sufficient nutrients for microbial growth, resulting in lower abundance of genes related to C degradation [10]. Additionally, C deficiency (as indicated by a low TC) might promote the C fixation activity of microorganisms at the early stage of revegetation [10]. This explains the substantial C fixation due to the higher abundance of *accA*, *acsA*, *pccA*, and *rbcL* in the 11-year-old site, which can be attributed to the efficient promotion of C fixation by microorganisms [57].

Soil N input generally increases during revegetation but a large amount of N could be taken up and used by plants, so the demand for soil microorganisms that regulate soil N cycling is greater [57]. The previous study showed a decrease in the relative abundance of N nitrification- and fixation-associated genes, whereas denitrification-related gene abundance increased with restoration years [10,59]. However, in our study, were observed an increase in the relative abundance of nitrification (*amoABC*) and ammonium assimilation (*ureC*) genes, whereas the abundance of denitrification (*narGHI* and *napAB*), DNRA (*narGHI* and *napAB*), and nitrification (*nxrAB*) first increased significantly and then decreased slightly with stand age (Table S2). This is in agreement with the findings of a previous study [59] and our hypothesis. With the increase of stand age, MBC and MBN increased by 3.4-fold and 3.0-fold (Table 1), respectively, and the proliferation of soil microorganisms increased their demand for N, thus increasing the abundance of related genes involved in the N cycle. These results suggest that stand age can increase the abundance of nitrification and denitrification genes, facilitating soil N cycling. In particular, the relative abundance of *gdhA* and *ureC* increased with stand age, which promoted the dynamic balance between urea metabolism and ammonia biosynthesis and, consequently, increased the availability and metabolic potential of soil N [60].

### 4.3. Linkages between Rhizosphere Soil Microbial and Environmental Factors

The soil microbial community composition was closely related to the abiotic and biotic factors along stand age (Figure 4). The soil microbial community composition in restored sites is mediated by the changes in soil properties such as AP, TN, C:N ratio, exchangeable irons, and enzyme activities [54,61]. In particular, the abundance and diversity of the bacterial community are significantly correlated with TC, TN, AN, and AP [62]. An et al. (2022) [61] found that TC, pH, and TP are the major abiotic factors driving the bacterial community structure in an arid oasis–desert ecotone. In our study, the RDA showed that the TC was the key abiotic factor affecting the bacterial community. This can be attributed to the fact that the bacterial community is dominated by Actinobacteria and Proteobacteria, and SOC is an important factor regulating their survival and development. Moreover, the soil bacterial community was also affected by the level of AP and AN [2]. However, in this study, AP and AN were not the main factors affecting the bacterial community, which was associated with a higher ALP activity and a higher relative abundance of Proteobacteria. A higher ALP promotes the release of soil P [60]. As the phylum Proteobacteria contains numerous N-fixing bacteria, a higher relative abundance of Proteobacteria increases the AN content [2]. In addition, N fixation by leguminous plants also contributes to a higher AN level. Consequently, this result suggests that although soil C content is rapidly increasing, it is still the main limiting factor for soil microbial community composition rather than N and P after revegetation of temperate deserts.

Soil fungi are considered a critical component of the soil and are involved in C and nutrient cycling; their functional composition is closely linked to the soil nutrient level [63]. Soil factors directly regulate the soil fungal diversity and community composition. Generally, soil pH is a primary driver of fungal community structure and function in a global context, and higher water availability may increase soil fungal community diversity and composition [64]. The previous large-scale study indicated that TN plays an important role in the soil fungal community [54,65]. In this study, TN and pH were the main abiotic factors driving the fungal community structure, which confirms previous findings [54,65]. With increasing stand age, the accumulation of organic matter of root exudates increases the nutrient level in the rhizosphere soil and changes the pH. The gradually decreasing soil pH changes the optimum range of the original fungal community, thereby affecting its composition. Specifically, the soil TN has a greater impact on pathotrophs in fungal communities, and pathotrophs prefer nutrient-rich environments [65]. However, N is the main factor limiting plant growth, thereby affecting microbial communities in terrestrial ecosystems [66]. In arid ecosystems, the limiting effect of N is more pronounced. Moreover, soil organic matter provides a substrate for the growth and reproduction of microorganisms. However, in our study, the N accumulation rate was slower than that of organic matter (the C:N ratio gradually decreased), which in turn aggravated N limitations.

The changes in the environmental factors also had a significant impact on the functional genes related to C and N cycling (Figure 7) [59]. In this study, the genes involved in C degradation and fixation were largely affected by the BG, which is in agreement with previous findings that the MBC and BG have positive effects on C cycling genes [10] and confirms the conclusion that soil microbial biomass and extracellular enzymes are the main factors driving C and N cycling [10,67]. The variations in the functional genes involved in N cycling during vegetation restoration were significantly correlated with the levels of TC and TN, as also observed in a previous study in a semiarid area [52]. Organic matter affects copiotrophs and oligotrophs and regulates soil microbial processes. In our study, there were changes in the soil properties in the restored sites as a result of plant colonization and community succession, and these changes may have shaped the microbial communities. In addition, biotic factors also largely contributed to the variations in the genes involved in C and N cycling (Figure 6). In particular, the MBN and TC had significant effects on N cycling genes, mostly because both MBN and TC are microbial metabolites as well as substrates and play an important role in microbial dissimilation and assimilation [10,52,68]. During revegetation, variations in microbial C and N cycling genes are frequently provoked by elevated levels of MBC, MBN, and extracellular enzyme activity. This implies that as the revegetation age increases, soil C sequestration may become more pronounced due to a reduction in C degradation function genes, while N cycling will be faster owing to more intense microbial activity induced by nutrient limitation.

Moreover, the changes in the composition and structure of microbial communities also affect soil biogeochemical cycling processes [10,69]. Previous studies found that soil microbial community composition shifted from oligotrophic groups (e.g., Acidobacteria, Chloroflexi) to copiotrophic groups (e.g., Actinobacteria, Proteobacteria, Bacteroidetes) during grassland restoration, which in turn affected the accumulation of soil carbon and the composition of the soil carbon pool [10]. Oligotrophic communities of soil microorganisms are mainly characterized by a survival strategy of low reproductive rate and high survival rate, and they are highly competitive and mainly degrade recalcitrant carbon as substrate. In contrast, copiotrophic communities were characterized by stronger cooperation between microorganisms, which mainly used reactive organic matter as a substrate, and were more conducive to the sequestration of recalcitrant carbon in the soil and the accumulation of a more stable carbon pool. In this study, the increase in the abundance of microorganisms of the phylum Antimicrobia and carbon fixation functional genes both suggest that the evolution of microbial communities influences soil carbon cycling processes during the restoration of shrub-dominated desert ecosystems, and may also have an important impact on the cycling processes of other elements.

## 5. Conclusions

Stand age significantly improved the abiotic and biotic factors of the rhizosphere soil, affecting the structure of the soil microbial community and the abundance of genes related to C and N cycling. The increase in stand age promoted soil N cycling by increasing the abundance of functional genes related to denitrification, DNRA, nitrification, and ammonium assimilation, whereas the capacity and potential of C cycling were reduced by decreasing the abundance of functional genes related to C degradation and increasing the abundance of C fixation genes. Soil abiotic and biotic factors had significant impacts on the composition of soil microbial communities and the abundance of functional genes involved in C and N cycling. The impact of stand age on the relative abundance of soil C and N cycling genes is mediated indirectly through the direct regulation of soil properties and microbial community structure. This suggests that soil microbial effects on C and N cycling depend on soil properties and microbial community composition. This study reveals the influence of stand age on microbial community structure and functional genes and the underlying mechanisms. It provides a basis for understanding the dynamics and response mechanisms of microorganisms during vegetation restoration as well as their role in C and N cycling.

## Figures and Tables

**Table 1:** Variations in rhizosphere soil abiotic and biotic factors along a chronosequence of stand age.

Properties		Stand Age (Year)			
		11 y	35 y	58 y	66 y
Abiotic factors	TN (g/kg)	0.23 ± 0.01 b	0.28 ± 0.03 b	0.31 ± 0.05 b	0.57 ± 0.03 a
	TC (g/kg)	3.86 ± 0.10 c	4.60 ± 0.14 b	4.56 ± 0.10 b	7.08 ± 0.15 a
	C:N	16.81 ± 0.70 a	16.82 ± 1.00 a	15.57 ± 2.38 a	12.39 ± 0.63 a
	AP (mg/kg)	72.21 ± 0.60 c	70.64 ± 0.29 c	78.92 ± 2.29 b	85.53 ± 0.20 a
	EC (μS/cm)	122.37 ± 2.38 c	148.87 ± 5.63 c	293.33 ± 2.19 b	717.33 ± 27.53 a
	pH	8.05 ± 0.02 b	8.11 ± 0.01 a	7.91 ± 0.01 c	7.78 ± 0.02 d
Biotic factors	MBC (mg/kg)	49.03 ± 1.18 c	65.28 ± 0.77 c	97.80 ± 1.12 b	164.38 ± 14.63 a
	MBN (mg/kg)	5.75 ± 0.48 d	8.54 ± 0.69 c	13.46 ± 0.69 b	17.41 ± 0.73 a
	BG (mg/g/h)	0.30 ± 0.01 a	0.12 ± 0.02 c	0.22 ± 0.04 b	0.22 ± 0.01 b
	ALP (μg/g/h)	35.03 ± 2.73 a	37.38 ± 5.44 a	31.10 ± 4.40 a	37.46 ± 1.70 a
	NAG (μmol/d/g)	5.35 ± 1.04 a	6.09 ± 0.52 a	6.62 ± 0.73 a	7.18 ± 1.58 a

Note: values are mean ± SE (*n* = 3). The different lowercase letters indicate significant differences at *p* < 0.05 level between the different stand age. TN, total nitrogen; TC, soil total carbon; AP, available phosphorus; EC, electrical conductivity; MBC, microbial biomass carbon; MBN, microbial biomass nitrogen; BG, *β*-1,4-glucosidase; ALP, alkaline phosphatase; NAG, *β*-N-acetylglucosaminidase.

## Data Availability

The raw data supporting the conclusions of this article will be made available by the authors on request.

## Supplementary Materials

The following supporting information can be downloaded at: https://www.mdpi.com/article/10.3390/microorganisms12091752/s1.
