# Supplementary material for: The Response of Rhizosphere Microbial C and N-Cycling Gene Abundance of Sand-Fixing Shrub to Stand Age Following Desert Restoration
Source: Microorganisms. 2024 Aug 23;12(9):1752. doi: 10.3390/microorganisms12091752 (PMC11434391; doi:10.3390/microorganisms12091752)
Supplement: Supplementary file 1 [file microorganisms-12-01752-s001.zip › Figure S1-S5 caption.docx]

Figure S1 The composition of soil microbial along stand age. (a), relative abundance of dominant bacterial genera; (b), relative abundance of dominant fungal genera; (c), NMDS of bacterial genera; (d), NMDS of fungal genera. “Other” includes genera after the top 20 in relative abundance ranking.

Figure S2 Shannon index of soil microbial along stand age at phylum level. (a), bacteria; (b), fungi.

Figure S3 The change of the relative abundance of soil microbial functional gene of carbon degradation with stand age.

Figure S4 Pearson’s correlation coefficients of environmental factors and carbon and nitrogen cycling functional genes.

Figure S5 Relationship between observed and latent variables. Ovals represent latent variables, rectangles represent observed variables, and numbers are correlation coefficients. (a), Soil properties included BG: *β*-1,4-glucosidase; MBN: microbial biomass nitrogen; NAG: *β*-N-acetyl glucosaminidase; (b), microbial community structure included community structure of bacteria and fungi by non-metric multidimensional scaling (NMDS); BNMDS: NMDS of bacteria; FNMDS: NMDS of fungi; (c), carbon cycling genes including the relative abundance of selected carbon degradation genes (cellulose, chitin, lignin, pectin); (d), nitrogen cycling genes including the relative abundance of genes related to assimilatory, dissimilatory, nitrogen fixation, organic nitrogen mineralization (Norg) processes.
