# Supplementary material for: The Response of Rhizosphere Microbial C and N-Cycling Gene Abundance of Sand-Fixing Shrub to Stand Age Following Desert Restoration
Source: Microorganisms. 2024 Aug 23;12(9):1752. doi: 10.3390/microorganisms12091752 (PMC11434391; doi:10.3390/microorganisms12091752)
Supplement: Supplementary file 1 [file microorganisms-12-01752-s001.zip › Highlights.docx]

**Highlights:**

- Stand age significantly changed the composition of the rhizosphere soil microbial community and the abundance of functional genes
- The alpha diversity of rhizosphere soil bacteria tended to increase with increasing stand age, whereas that of fungi decreased
- The bacterial community was regulated by MBC, MBN, and TC, whereas that of fungal community driven by TN, EC, pH, and MBC
- Stand age enhance the function of N cycling, while decrease C cycling functional genes
- The stand age regulated C and N cycling functional genes through soil properties and microbial community structure
