# Supplementary material for: The Response of Rhizosphere Microbial C and N-Cycling Gene Abundance of Sand-Fixing Shrub to Stand Age Following Desert Restoration
Source: Microorganisms. 2024 Aug 23;12(9):1752. doi: 10.3390/microorganisms12091752 (PMC11434391; doi:10.3390/microorganisms12091752)
Supplement: Supplementary file 1 [file microorganisms-12-01752-s001.zip › Supplementary Table S1-S3.docx]

**Supplementary Table S1** Relative abundance of microbial C fixation genes during vegetation restoration in the rhizosphere soil. Different letters indicate significant differences among different stand ages in each kind of functional genes based on one-way ANOVA (LSD test) at *p* < 0.05.

| Gene | Stand age (y) | | | | |
| --- | --- | --- | --- | --- | --- |
|  | 11y | 35y | 58y | 66y |  |
| *accA* | 123.00±5.39a | 80.31±6.84bc | 86.65±18.01b | 48.61±8.39c |  |
| *aclA* | 13.82±0.23b | 33.61±1.90a | 12.95±2.48b | 26.02±5.71a |  |
| *acsA* | 22.46±2.63a | 13.46±1.49a | 15.36±6.30a | 26.85±4.26a |  |
| *frdA* | 588.34±34.41b | 1028.43±46.86a | 614.68±112.96b | 882.13±48.33a |  |
| *korA* | 176.14±18.98b | 304.27±11.46a | 201.38±52.84b | 226.86±15.82ab |  |
| *mcr* | 13.50±3.91a | 19.95±8.45a | 25.93±9.33a | 18.86±9.49a |  |
| *mct* | 5.69±2.95b | 23.64±5.33a | 15.98±0.82ab | 5.64±5.64b |  |
| *pccA* | 97.42±13.93a | 64.17±9.15a | 78.99±19.19a | 53.11±12.30a |  |
| *rbcL* | 109.19±9.29a | 104.22±19.68a | 99.03±17.80a | 81.76±3.97a |  |
| *sucD* | 590.36±35.61b | 896.04±66.94a | 666.69±103.30ab | 767.03±57.58ab |  |

**Supplementary Table S2** Relative abundance of microbial N cycling genes during vegetation restoration in the rhizosphere soil. Different letters indicate significant differences among different stand ages in each kind of functional genes based on one-way ANOVA (LSD test) at *p* < 0.05.

| Gene | Stand age (y) | | | | |
| --- | --- | --- | --- | --- | --- |
|  | 11y | 35y | 58y | 66y |  |
| *amoABC* | 83.67±18.19c | 314.54±24.43b | 140.85±31.87c | 407.16±24.62a |  |
| *gdhA* | 72.07±9.97a | 95.82±6.16a | 103.45±17.56a | 111.05±9.59a |  |
| *narB & NR & nasAB* | 43.01±5.87a | 36.22±7.11a | 38.95±13.54a | 31.64±4.99a |  |
| *narGHI&napAB* | 80.33±8.84c | 201.85±7.80a | 104.79±23.29bc | 178.75±21.51ab |  |
| *nifDKH&vnfDKGH* | 16.66±4.50a | 19.31±5.75a | 7.23±5.51a | 11.32±7.53a |  |
| *nirBD & nrfAH* | 200.81±4.21a | 206.96±17.11a | 152.71±30.30a | 214.99±8.77a |  |
| *nirK & nirS* | 4.08±4.08a | 11.21±6.43a | 1.90±1.08a | 5.71±5.71a |  |
| *NIT-6 & nirA* | nc | 1.05±1.05 | 2.41±2.41 | nc |  |
| *norBC* | 1.83±0.18a | 0.83±0.83a | 2.01±2.01a | 0.46±0.46a |  |
| *nosZ* | nc | nc | 1.44±1.44 | nc |  |
| *nxrAB* | 74.56±8.14c | 200.14±9.14a | 101.13±25.41bc | 176.52±21.96ab |  |
| *ureC* | 273.11±4.42b | 395.59±36.73ab | 332.78±39.53ab | 412.04±12.17a |  |

“nc’ mean no checked genes.

**Supplementary Table S3** Variation explained of abiotic factors (TC, TN, AP, EC, pH) and biotic factors (MBC, MBN, BG, ACP, NAG) to the carbon cycling and nitrogen cycling gene groups. A, abiotic factors; B, biotic factors; C, interaction of A and B.

| Gene groups | | Tested fraction | | |  | Group members | |
| --- | --- | --- | --- | --- | --- | --- | --- |
|  |  | A+B+C | A+C | B+C |  | A | B |
| Carbon and nitrogen cycling | *F* | 1.9 | 1.9 | 1.8 |  | TC, pH, TN | BG, MBC, NAG |
|  | *P* | 0.002 | 0.002 | 0.002 |  |  |  |
| Carbon degradation | *F* | 1.5 | 1.5 | 1.9 |  | TC, pH, TN | BG, MBC, MBN |
|  | *P* | 0.006 | 0.012 | 0.002 |  |  |  |
| Carbon fixation | *F* | 2.4 | 2.4 | 2.3 |  | TC, pH, TN | BG, MBC, NAG |
|  | *P* | 0.008 | 0.004 | 0.008 |  |  |  |
| Nitrogen cycling | *F* | 2.3 | 3.1 | 1.6 |  | TN, pH, TC | MBC, BG, NAG |
|  | *P* | 0.01 | 0.002 | 0.136 |  |  |  |
