# Supplementary figures and images for: The Response of Rhizosphere Microbial C and N-Cycling Gene Abundance of Sand-Fixing Shrub to Stand Age Following Desert Restoration
Source: Microorganisms. 2024 Aug 23;12(9):1752. doi: 10.3390/microorganisms12091752 (PMC11434391; doi:10.3390/microorganisms12091752)

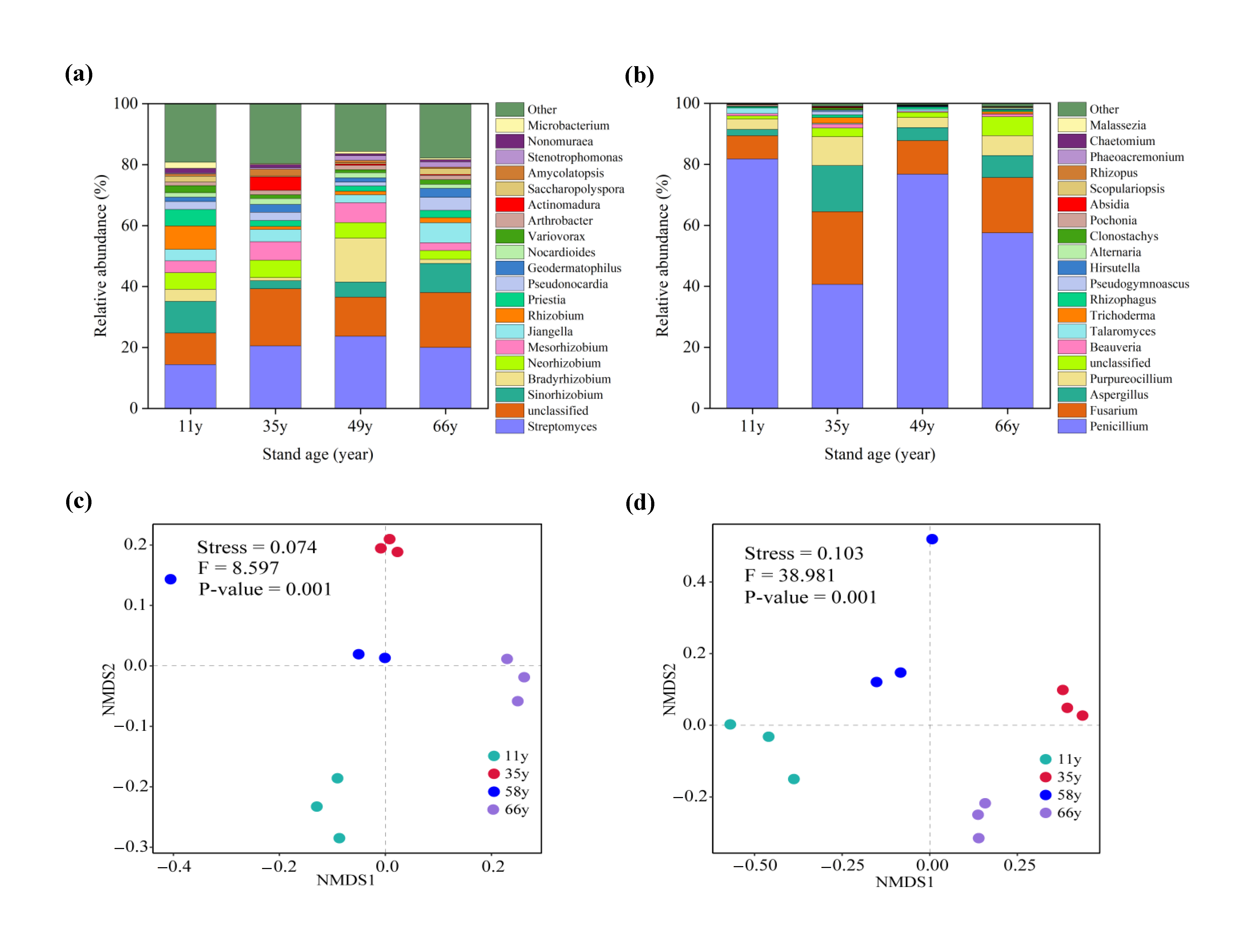

Supplement: Supplementary file 1 [file microorganisms-12-01752-s001.zip › Figure S1.tiff]

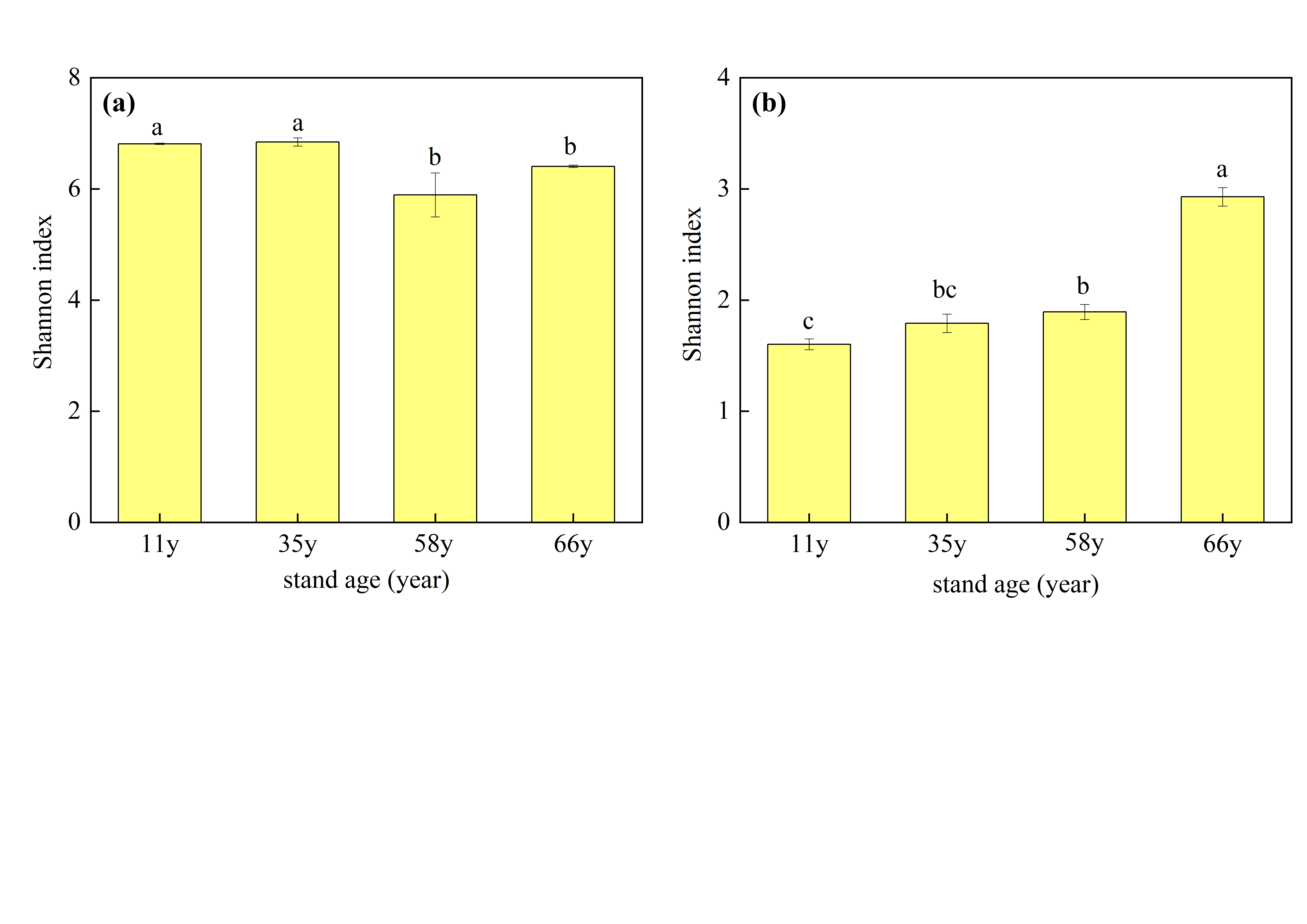

Supplement: Supplementary file 1 [file microorganisms-12-01752-s001.zip › Figure S2.tif]

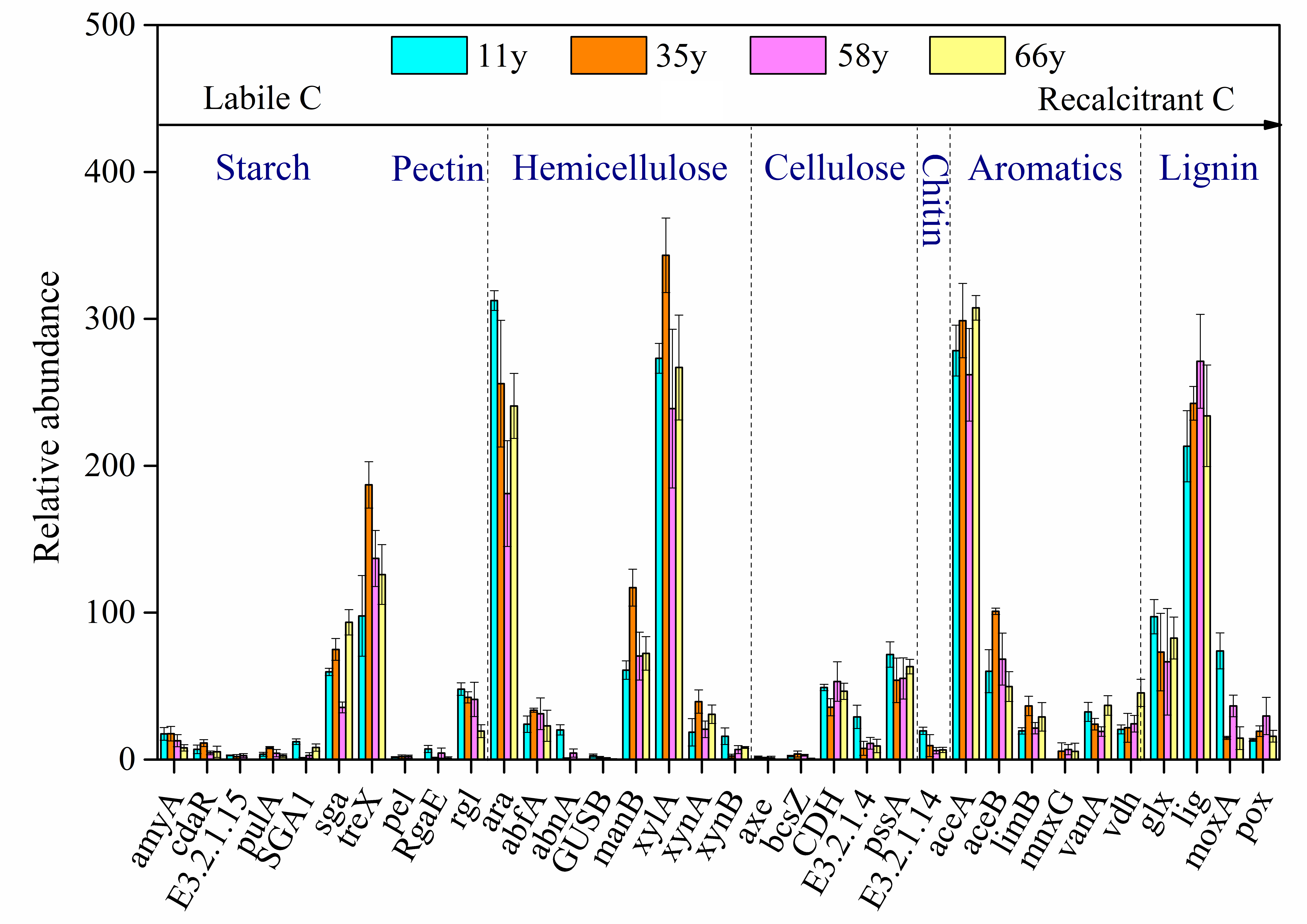

Supplement: Supplementary file 1 [file microorganisms-12-01752-s001.zip › Figure S3.tif]

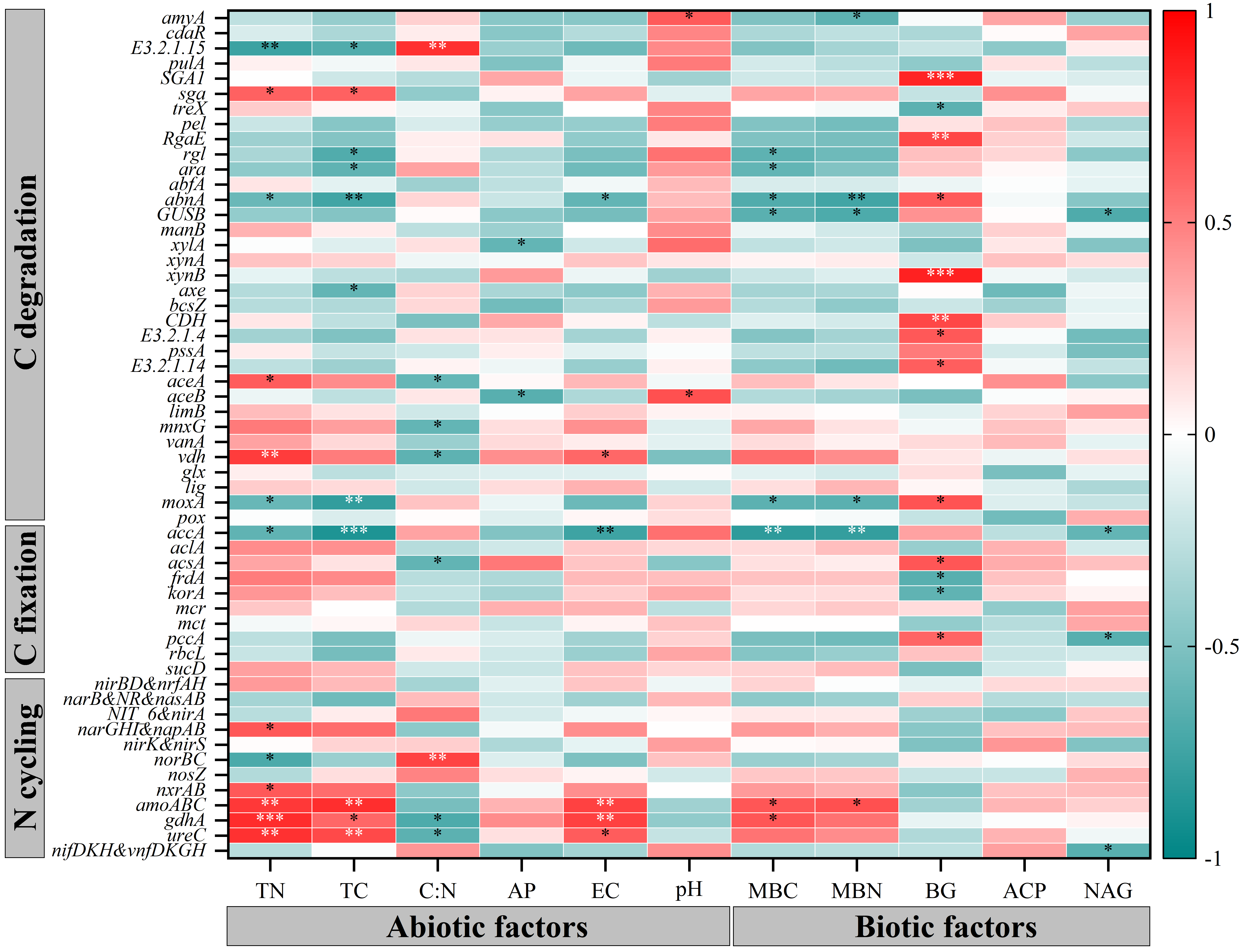

Supplement: Supplementary file 1 [file microorganisms-12-01752-s001.zip › Figure S4.tif]

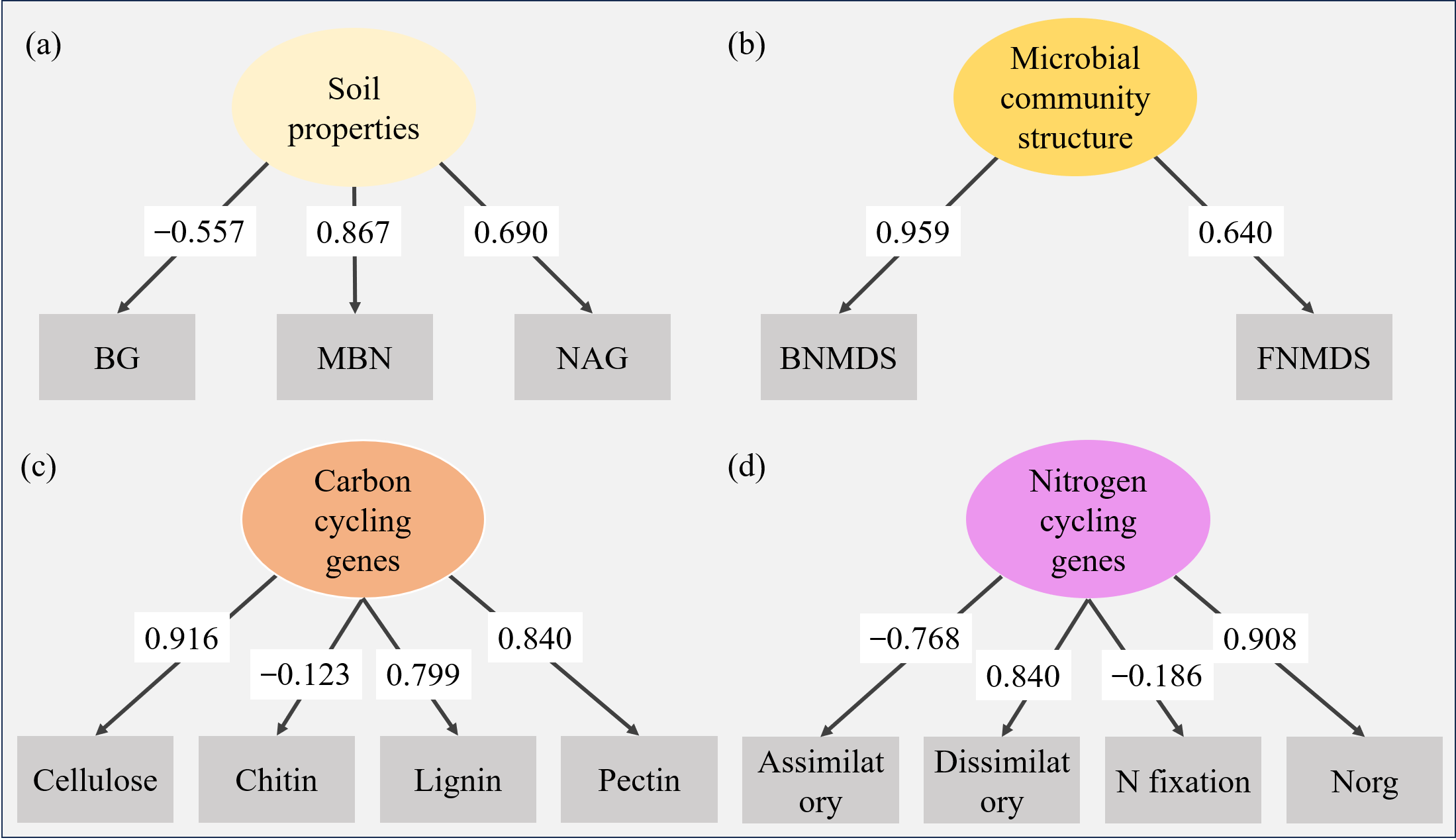

Supplement: Supplementary file 1 [file microorganisms-12-01752-s001.zip › Figure S5.tif]
